# Epigenomics and Lipidomics Integration in Alzheimer Disease: Pathways Involved in Early Stages

**DOI:** 10.3390/biomedicines9121812

**Published:** 2021-12-02

**Authors:** Carmen Peña-Bautista, Lourdes Álvarez-Sánchez, Antonio José Cañada-Martínez, Miguel Baquero, Consuelo Cháfer-Pericás

**Affiliations:** 1 Alzheimer’s Disease Research Group, Health Research Institute La Fe, 46026 Valencia, Spain; mariadelcarmen_pena@iislafe.es (C.P.-B.); lourdes_alvarez@iislafe.es (L.Á.-S.); baquero_miq@gva.es (M.B.); 2 Division of Neurology, University and Polytechnic Hospital La Fe, 46026 Valencia, Spain; 3 Data Science Unit, Health Research Institute La Fe (IIS La Fe), 46026 Valencia, Spain; bioestadistica@iislafe.es

**Keywords:** Alzheimer disease, plasma, biomarker, lipids, microRNAs, integration

## Abstract

Background: Alzheimer Disease (AD) is the most prevalent dementia. However, the physiopathological mechanisms involved in its development are unclear. In this sense, a multi-omics approach could provide some progress. Methods: Epigenomic and lipidomic analysis were carried out in plasma samples from patients with mild cognitive impairment (MCI) due to AD (*n* = 22), and healthy controls (*n* = 5). Then, omics integration between microRNAs (miRNAs) and lipids was performed by Sparse Partial Least Squares (s-PLS) regression and target genes for the selected miRNAs were identified. Results: 25 miRNAs and 25 lipids with higher loadings in the sPLS regression were selected. Lipids from phosphatidylethanolamines (PE), lysophosphatidylcholines (LPC), ceramides, phosphatidylcholines (PC), triglycerides (TG) and several long chain fatty acids families were identified as differentially expressed in AD. Among them, several fatty acids showed strong positive correlations with miRNAs studied. In fact, these miRNAs regulated genes implied in fatty acids metabolism, as elongation of very long-chain fatty acids (ELOVL), and fatty acid desaturases (FADs). Conclusions: The lipidomic–epigenomic integration showed that several lipids and miRNAs were differentially expressed in AD, being the fatty acids mechanisms potentially involved in the disease development. However, further work about targeted analysis should be carried out in a larger cohort, in order to validate these preliminary results and study the proposed pathways in detail.

## 1. Introduction

Alzheimer disease (AD) is the most prevalent dementia [1]. Some hallmarks are clearly related to AD; accumulation of extracellular β-amyloid plaques and intracellular Tau neurofibrillary tangles. Nevertheless, the physiopathological mechanisms involved in the complex and multifactorial AD development remain unclear [2]. Therefore, a multi-omics approach could provide some progress in this field.

AD development could involve the reconfiguration of the epigenome and the modification of some genes expression have an impact in different disease pathways [3]. Specifically, differential expression of microRNAs have been found in recent AD studies [4,5]. These miRNAs could act as an epigenetic mechanism modifying the expression of different proteins post-transcriptionally [6]. Therefore, an increase or decrease in the levels of miRNAs could influence the expression of different proteins or enzymes. In this context, Hébert et al. described different miRNAs related to Amyloid precursor protein (APP) expression [7]. Therefore, epigenomics could be implicated in this pathology.

Lipidomics could also play an important role in AD development. In fact, lipids, the main component of cell membranes, are strongly related to brain function and neurodegenerative diseases [8]. Specifically, the lipids from phospholipids, triglycerides, sphingolipids and cholesteryl esters correlated with clinical AD diagnosis, brain atrophy and disease progression [9]. A previous study developed a combination of 24 molecules to classify patients with high accuracy (>70%), and identified some metabolic features (triglycerides, phosphatidylcholines) [10].

Integrative network analysis of multi-omics results allowed us to identify molecular mechanisms in AD. A previous study based on RNA and Whole Genome Sequencing (WGS) observed signaling circuits of complex molecular interactions in key brain regions [11]. In another multi-omics study, Xicota et al. 2019 studied RNAseq, metabolomics and lipidomics, they found a signature of some blood metabolites and transcripts, which identified asymptomatic AD patients [12]. Additionally, a study from the literature showed the integration of genome-wide association studies with expression data, identifying some genes related to AD physiopathology. Specifically, the pathways were involved in calcium homeostasis [13]. In addition, a recent study was based on an integrative analysis of blood microRNAs expression and genomic data to develop an AD prognosis model, including 24 single nucleotide polymorphism-microRNA (miR-eQTLs), as well as age, sex, and APOE4 genotype [14]. From these miR-eQTLs, four genes related to AD (SHC1, FOXO1, GSK3B, and PTEN) were identified. Similarly, a genomics and metabolomics study demonstrated the utility of these data integration with AD risk factors to understand the mechanisms involved, revealing the importance of glycine as a mediator in cardiovascular and diabetes risk [15]. Epigenomic–lipidomic integration would allow the global study of the regulatory mechanisms involved in AD such as lipid homeostasis, oxidative stress, synaptic vesicle trafficking, inflammation, etc. [16]. These omics data were analysed together to develop an understanding of lipid regulation by epigenomics. Previous works based on the analysis of genome-wide DNA methylation showed that an epigenetic pattern was associated with cholesterol regulation [17]. In addition, in Parkinson Disease (PD), an epigenetic (DNA methylation) regulation was involved in the inactivation of the autophagy system, contributing to protein accumulation [18]. Thus, the study of the integration between epigenomics and lipidomics could show lipid regulation mechanisms involved in AD.

The aim of this work was to carry out the integration of epigenomics and lipidomics analysis in plasma samples from patients with mild cognitive impairment (MCI) due to AD, in order to advance the knowledge of early physiopathological mechanisms.

## 2. Materials and Methods

### 2.1. Participants and Samples Collection

All the participants were aged between 50 and 80 years old. Patients with known major neurological or psychiatric conditions were excluded. Assessment included a neuropsychological evaluation (Repeatable Battery for Assessment of Neuropsychological Status (RBANS) [19], Functionality Assessment Questionnaire (FAQ) [20], Clinical Dementia Rating (CDR) [21], MMSE [22]), analysis by means of NMR-TAC and cerebrospinal fluid (CSF) levels of amyloid β-42 peptide, *t*-Tau and *p*-Tau (Table 1). According to this, participants were classified into patients with MCI-AD (*n* = 22), and healthy controls (*n* = 5).

Blood samples from participants were collected into EDTA-tubes, and plasma was separated. Then, plasma samples were stored at −80 °C until the analysis.

### 2.2. Omics Analysis

#### 2.2.1. Epigenomics

Epigenomic analysis was carried out by means of NGS NextSeq 550 platform (Illumina, San Diego, CA, USA) by single read sequencing of 50 cycles (1 × 50 bp). Data were processed and normalised to quantify and generate miRNA counts. The miRbase (v.21) allowed us to identify the miRNAs. Then, the identification of potential target genes for the selected miRNAs were carried out by miRbase (v.21, Manchester, UK).

#### 2.2.2. Lipidomics

Lipidomic analysis was carried out by means of ultra-performance liquid chromatography coupled to time-of-flight mass spectrometry (MS). The internal standard consisted of a mix of: MG(17:0), LPC(17:0), Cer(d18:1/17:0), DG(17:0/17:0), SM(d18:1/17:0), PE(17:0/17:0), PC(17:0/17:0), TG(17:0/17:0/17:0), CE(17:0), PG(17:0/17:0) and PS(17:0/17:0). The chromatographic and mass spectrometry conditions were those established in the standard procedures of the Analytical Unit from Health Research Unit from Health Research Institute La Fe. Briefly, data were processed for peak detection, noise filtering, and peak alignment. The procedure was conducted to reduce the intra-batch variability, as well as to ensure the quality and reproducibility of the analysis. It consisted of a random injection order, at the beginning of the sequence 5 quality control (QC) samples were analysed in order to condition column and equipment, and every 5–7 samples a QC was analysed in Full MS mode. Additionally, at the beginning, middle, and end of the sequence, some QCs were analysed in Fragmentation in Data Independent mode and in Fragmentation in Data Dependent mode to proceed to the annotations of lipid species with the LipidMS annotations package. Then, data were filtered to exclude variables whose coefficients of variation in the QCs were higher than 30%, and variables with zeros in more than 60% of samples. Then, data were normalised. Finally, the library LipidMSid was used to identify the lipids.

### 2.3. Statistical Analysis and Lipidomics-Epigenomics Integration

Data were summarised using median (1st, 3rd quartiles) for quantitative variables and absolute frequency (%) for qualitative variables.

Sparse Partial Least Squares (sPLS) regression was applied to the previous data sets to select variables (miRNAs, lipids) and integrate them. The sPLS approach combines both integration and variable selection on two data sets in a one-step strategy [23].

Then, the graphical representations (correlation circle plots, heatmaps, relevance networks) resulting from the statistical approach were plotted.

Individual differences between groups were carried out by Mann–Whitney test, and correlations by Pearson Correlation. In all the cases, statistical significance was fixed in a *p* value of 0.05.

Statistical analyses were performed using R software (v 4.0.3, Auckland, CA, USA) and mixOmics (v 6.16.2) and clickR (v 0.7.35) packages and SPSS software version 20.0 (SPSS, Inc., Chicago, IL, USA).

## 3. Results

### 3.1. Participants

Table 2 shows the demographic and clinical data for the participants. As expected, CSF biomarkers levels and neuropsychological tests were different between groups. In fact, the MCI-AD group showed lower levels for amyloid β-42, and higher levels for *t*-Tau and *p*-Tau; also, MCI-AD group showed lower scores for MMSE, and RBANS, and higher scores for CDR and FAQ.

### 3.2. Omics Integration

The sPLS model integrated two data matrices X (epigenomics) and Y (lipidomics). Additionally, sPLS performed simultaneous variables selection in the two data sets, by means of LASSO penalization on the pair of loading vectors. In this sense, two components were chosen, and 25 variables were selected on each dimension and for each data set. The X-block represented miRNAs, and the Y-block represented lipids.

**Figure 1:**
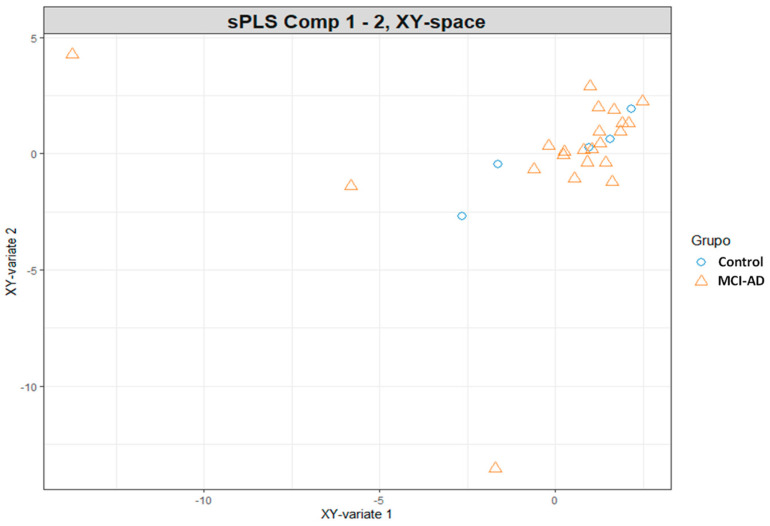
Scatter plot for participants samples in sPLS analysis. Represent the samples distribution in the ‘common’ subspace between the two sets of components (epigenomics and lipidomics variables).

Samples from both sets were represented in the ‘common’ subspace spanned by the principal components (PC1, PC2). As can be seen in Figure 1, samples were differentiated in the plot according to the participants group, there was not observed a clear separation. 

**Figure 2:**
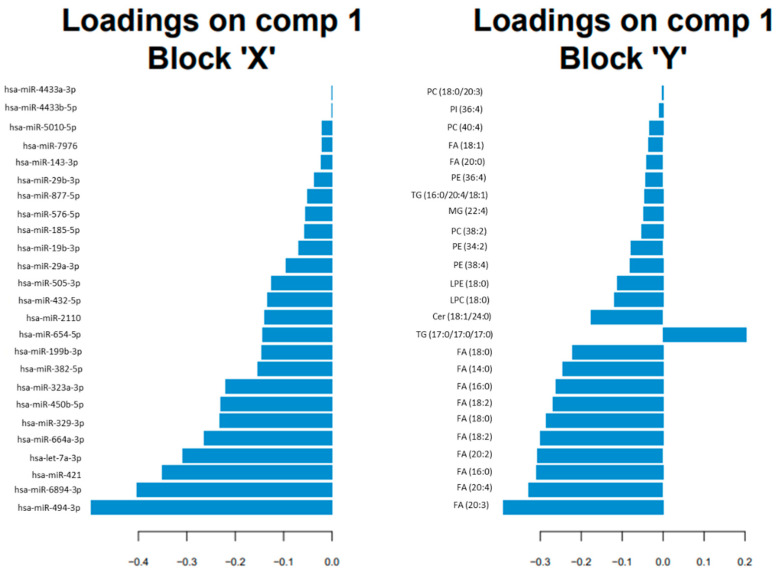
Horizontal barplot to visualise loading vector. The contribution of each variable for each component (comp) is represented in a barplot, where each bar length corresponds to the loading weight (importance) of the feature. The loading weight can be positive or negative.

Among the 25 selected variables for each data set, the miRNAs (block X) with higher loadings in the sPLS regression were hsa-miR-494-3p, hsa-miR-6894-3p, hsa-miR-421 and hsa-let-7a-3p; and the lipids (block Y) with higher loadings were FA (20:3), FA (20:4), FA (16:0), FA (20:2), and FA (18:2) (see Figure 2).

The correlation circle plot depicted microRNAs and lipids selected on each component. As can be seen in the Supplementary Material (Figure S1), some subsets of variables were important to define each component. Actually, some miRNAs (hsa-miR-5010-5p, hsa-miR-421, hsa-miR-664a, hsa-miR-29b-3p, hsa-let-7a-3p, hsa-miR-19b-3p) and some lipids (FA (20:4), FA (20:3), FA (18:0)) mainly participated in defining the sPLS component 2; and some miRNAs (hsa-miR-335-3p, hsa-miR-532-3p, hsa-miR-379-5p, hsa-miR-4646-3p, hsa-miR-425-3p) mainly participated in defining component 1. Additionally, miRNAs, such as hsa-miR-421 and hsa-miR-5010-5p, were positively correlated to the lipids FA (20:4) and FA (20:3); while these miRNAs were negatively correlated to the lipid TG (17:0/17:0/17:0).

**Figure 3:**
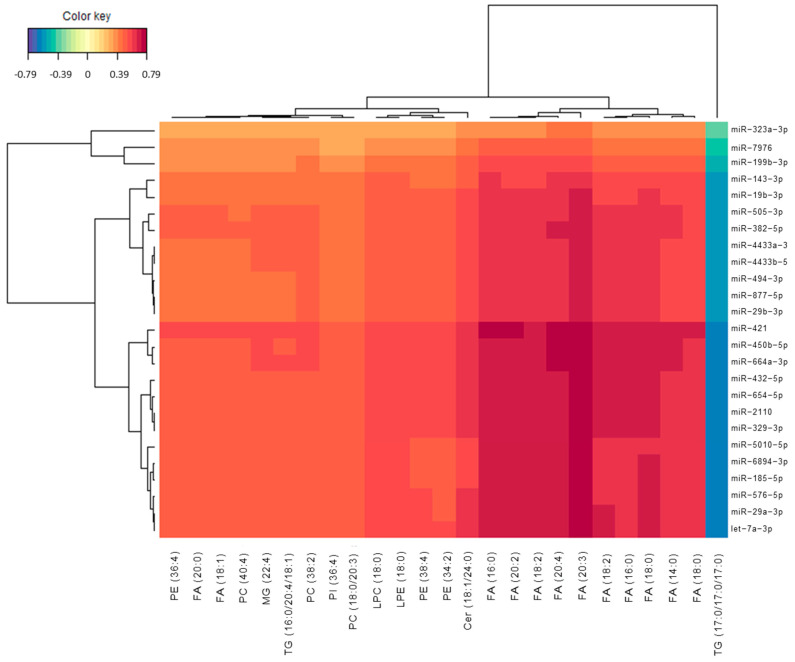
Heatmap representing correlations between miRNAs and lipid variables. Red colour represents positive correlations and blue colour represents negative correlations.

**Figure 4:**
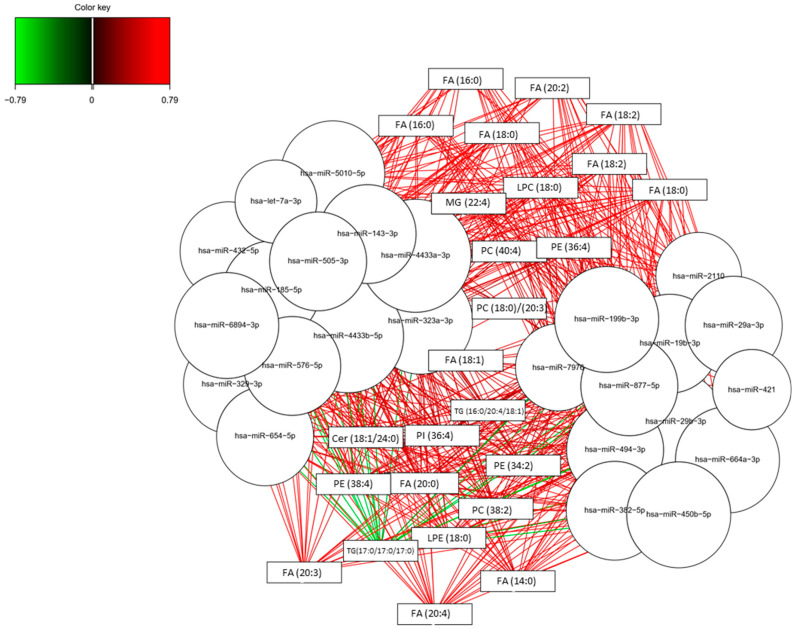
Relevance associations network for sPLS. Pair-wise similarity matrix directly obtained from the latent components was calculated. The similarity value between a pair of variables is obtained by calculating the sum of the correlations between the original variables and each of the latent components of the model. The values in the similarity matrix can be seen as a robust approximation of the Pearson correlation.

The integration results were depicted by means of a heatmap. The similarity matrix was obtained from the sPLS results [24], and agglomerative hierarchical clustering was derived using the Euclidean distance as the similarity measure, and the Ward methodology [25]. In this sense, Figure 3 shows the heatmap for the correlations between miRNAs and lipids selected from sPLS. The red colour corresponded to positive correlation, while the blue colour corresponded to negative correlation. Most of the correlations were positive. In general, Figure 4 showed a positive correlation between studied miRNAs and lipids. However, the lipid TG (17:0/17:0/17:0) showed a negative correlation with all the described miRNAs. In addition, similar miRNAs were grouped, showing clusters for miR-29a-3p, let-7a-3p, miR-576-5p, miR-185-5p, miR-6894-3p, miR-5010-5p; for miR-29b-3p, miR-877-5p, miR-494-3p, miR-4433a-3p, miR-4433b-5p; and for miR-421, miR-450b-5p, miR-664a-3p, miR-432-5p, miR-654-5p, miR-2110, miR-329-3p. In addition, similar lipids were grouped, showing clusters for FA (18:0)/FA (14:0)/FA (18:0)/FA (16:0)/FA (18:2) and FA (20:3)/FA (20:4)/FA (18:2)/FA (20:2)/FA (16:0).

### 3.3. Potential Pathways Involved in AD

In Table 3, the predicted target genes for the selected miRNAs were described paying special attention to the genes that are implied in lipid metabolism, specifically in fatty acids pathways, which showed correlation with the miRNAs. In fact, fatty acids family showed the strongest correlations with miRNAs (see Figure 4). Among the identified target genes, several enzymes, such as elongases (ELOVL1, ELOVL2, ELOVL3, ELOVL4, ELOVL5, ELOVL6, ELOVL7), fatty acid desaturase (FADS6), fatty acyl-CoA reductases (FAR 1, FAR 2), fatty acid binding protein (FABP7), and fatty acid 2-hydroxylase (FA2H) were highlighted.

Another representation for the integration results is based on relevance network for sPLS regression, showing simultaneously positive and negative correlations between the two variable types (microRNAs, lipids). As can be seen in Figure 4 and the Supplementary Material in Table S1, most of these correlations were positive. Specifically, the highest positive correlations corresponded to these pairs of variables (FA (16:0) and FA (20:2) with hsa-miR-664, hsa-miR-432, hsa-miR-421, and hsa-miR-450b-5p; FA (18:0) and FA (18:2) with hsa-miR-664, hsa-miR-421 and hsa-miR-450b-5p; FA (20:3) and FA (20:4) with hsa-miR-664, hsa-miR-211, hsa-miR-432, hsa-miR-329, hsa-miR-654, hsa-let-7a-3p, hsa-miR-29a-3p, hsa-miR-421, and hsa-miR-450b-5p). On the other hand, the highest negative correlations corresponded to the lipid TG (17:0/17:0/17:0) with some miRNAs (hsa-miR-664-3p, hsa-miR-2110, hsa-miR-432-5p, hsa-miR-329-3p, hsa-miR-654-5p, hsa-miR-185-5p, hsa-let-7a-3p, hsa-miR-576-5p, hsa-miR-29a-3p, hsa-miR-6894-3p, hsa-miR-421, hsa-miR-450b-5p).

### 3.4. Lipidomics and Epigenomics in AD

From the univariate analysis, differences between groups were not obtained for miRNAs nor individual lipids. Median values are summarised as Supplementary Material (Table S2). In addition, boxplots representing the lipid levels for each participants group were also depicted in the Supplementary Material (Figure S2).

In addition, the analysis between age/gender and biomarkers levels showed no correlations for any miRNA or lipid analysed.

## 4. Discussion

Epigenomics and lipidomics analyses were carried out in plasma samples from early AD patients, identifying microRNAs and lipids, respectively. From these results, integration analysis was carried out in order to study associations between both compounds families; to evaluate their potential relationship with early AD development; and identify the potential pathways altered in early stages of the disease.

Some studies in literature are focused on multi-omics integration, mainly based on proteomics and miRNAs [26]. However, few studies are focused on lipidomic and miRNAs integration, which allow us to identify different biological activities involved in cell communication [27]. In general, the integration of omics results (lipidomics, metabolomics, proteomics, epigenomics) helps to give a global image of the mechanisms involved in complex diseases [28]. Nevertheless, this field of research is still underdeveloped in AD and few studies are based on this integration [16].

In the present study, integration and selection of variables from each dimension showed that some microRNAs (hsa-miR-494-3p, hsa-miR-6894-3p, hsa-miR-421 and hsa-let-7a-3p) and some lipids (FA (20:3), FA (20:4), FA (16:0), FA (20:2), FA (18:2)) had higher loadings in the regression model. Similarly, a previous study carried out in plasma from amyloid positive and amyloid negative participants obtained a signature of 71 miRNAs differentially expressed between groups, highlighting the hsa-miR-421 and hsa-let-7a-3p [29]. In addition, a previous study from Hojati et al. revealed that hsa-miR-494-3p was slightly up-regulated in AD patients and that it was related to metabolic and cellular response to stress pathways [30]; while Lv et al., found that levels of hsa-let-7a-3p were elevated in patients with early onset familiar AD [31]. The up-regulation of hsa-let-7a-3p showed an increase in neurotoxicity in AD cell model [32]. On the other hand, previous studies found several fatty acids levels increased or decreased in AD [33,34]. Specifically, AD was related to lower levels of myristic 14:0, palmitic 16:0, stearic 18:0 and oleic 18:1 acid and a higher proportion of linoleic acid 18:2n−6 [33]. However, this study was limited to FAs from 14:0 to 22:6 and did not determine all lipidic profiles. In addition, Conquer et al. described lower levels of phospholipid, PC 20:5n-3, DHA, total n−3 fatty acids, the n−3/n−6 ratio and phospholipid 24:0 compared to controls [34]. Moreover, Conquer et al. did not find differences for FA (20:3), FA (20:4), FA (20:2) and FA (18:2) in plasma samples from AD, cognitive impairment, and patients with other neurodegenerative diseases [34]. This discrepancy with the present results could be due to differences in AD diagnosis methods, since the previous study did not use CSF biomarkers to identify AD patients. In fact, these participants were classified by amyloid PET, and biomarkers were measured in erythrocytes. In addition, erythrocyte fatty acid composition varied according to disease development, showing differences between AD and non-AD participants for FA (20:4) but not for FA (20:3), FA (20:2) nor FA (18:2) [35].

Regarding correlations between microRNAs and lipids, and similarities among them in each omics data group, they showed that most of these correlations were positive. However, previous studies that correlated epigenomics (DNA hydroxymethylation) and metabolomics showed more variety between positive and negative correlations [36]. More specifically, several studies in neurodegeneration revealed the interaction between miRNAs expression and lipids regulation, mainly focussed on cholesterol metabolism [37]. Jauouen et al. described miR-33 function modulating ABCA1 and interfering with Aβ plaque formation through cholesterol metabolism regulation [38]. In the present study, some miRNAs (miR-29a-3p, let-7a-3p, miR-576-5p, miR-185-5p, miR-6894-3p, miR-5010-5p; for miR-29b-3p, miR-877-5p, miR-494-3p, miR-4433a-3p, miR-4433b-5p; for miR-421, miR-450b-5p, miR-664a-3p, miR-432-5p, miR-654-5p, miR-2110, miR-329-3p) were grouped reflecting their similarity. Taking into account previous works, Kumar et al. found different miRNAs clustered expression, differentiating AD and control participants (hsa-miR-4741, hsa-miR-4668-5p, hsa-miR-3613-3p, hsa-miR-5001-5p, miR-4674) [39]. The discrepancies with present results may be due to the difference in the diagnosis of the patients, since the study from Kumar et al. was not based on CSF biomarkers. Moreover, Denk et al. showed clustered expression of miRNAs in control, AD and frontotemporal dementia participants, showing that some clusters included miRNAs from the same family, while others included different families in the same cluster, as in the present study [40]. However, the set of analysed miRNAs was limited. On the other hand, some lipids were grouped in the present paper (FA (18:0)/FA (14:0)/FA (18:0)/FA (16:0)/FA (18:2); FA (20:3)/FA (20:4)/FA (18:2)/FA (20:2)/FA (16:0)). In this sense, previous findings in an AD mice model showed different lipids expression clusters along the disease progression (two, three, seven months), showing mainly PEs in two months progression and a predomination of TG at seven months [41]. In addition, Kumar et al. described the co-regulation of different lipid sets, among which 17 were fatty acids [42].

Finally, the highest positive correlations between microRNAs and lipids were mainly for hsa-miR-664, hsa-miR-432, hsa-let-7a-3p, hsa-miR-29a-3p, hsa-miR-421 and hsa-miR-450b-5p with some fatty acids (FA (16:0), FA (18:0), FA (20:2), FA (20:3), FA (20:4)). In general, the described miRNAs showed a positive correlation with fatty acids. Of note, these miRNAs targeted sequences in genes implied in fatty acids metabolism. In this sense, previous studies showed a relationship between AD and fatty acids metabolism, demonstrating differential levels of fatty acids (FA (16:0), FA (18:0), FA (18:1), FA (18:2), FA(20:4), FA (20:5), FA (22:6)) similar to the present results [43]. Regarding hsa-miR-421, it showed a positive correlation with some detected lipids (FA (16:0), FA (20:2), FA (18:2), FA (20:4), FA (20:3), FA (18:0), FA (14:0)). Previous works identified the relationship between this miRNA and lipid metabolism regulation, specifically with triacylglycerol levels [44].On the other hand, the highest negative correlations corresponded to the triglyceride (TG (17:0/17:0/17:0)) with some miRNAs (hsa-miR-664-3p, hsa-miR-432-5p, hsa-miR-329-3p, hsa-miR-654-5p, hsa-miR-185-5p, hsa-let-7a-3p, hsa-miR-576-5p, hsa-miR-29a-3p, hsa-miR-421, hsa-miR-450b-5p). Similarly, in literature it was shown that hsa-miR-29a could regulate the lipoprotein lipase (LPL) that catalyses hydrolysis of the triglycerides [45].

The main limitation of this study is the reduced number of healthy control patients. However, the availability of biologically identified (CSF biomarkers) patients with MCI due to AD provides a great potential in the identification of potential pathways involved in early AD. Other limitations in this study are: (i) the analytical method is a semiquantitative approach, (ii) the ApoE genotype has not been taken into account, although it is known that ApoE is involved in lipid homeostasis.

## 5. Conclusions

The present study highlights the potential of a multi-omics approach in the development of a signature of biomarkers of MCI-AD, as well as the description of potential metabolic pathways involved in AD since its early stages. Specifically, epigenomics and lipidomics integration allowed us to identify some associations between microRNAs and lipids, showing their relationship with early AD development. In fact, fatty acids impairment could be an important pathway involved in early AD. However, further work based on targeted analysis should be carried out in a larger cohort in order to validate these preliminary results, as well as to study the proposed pathways in detail.

## Figures and Tables

**Table 1:** Clinical and neuropsychological criteria for participants’ classification.

Test or Biomarker	Participant Group	
	Control	MCI-AD
CDR *	0–0.5	0.5–1
MMSE *	≥27	<27
RBANS.DM *	≥85	<85
FAQ	<9	>9
Neuroimaging Structural (NMR-TAC)	Normal	Altered
CSF amyloid β42 (pg mL^−1^)	≥700	<700
CSF *t*-Tau (pg mL^−1^)	<350	≥350
CSF *p*-Tau (pg mL^−1^)	<85	≥85

* In MCI-AD group minimum 2 of the 3 cognitive status test (CDR, MMSE, RBANS.DM) should be altered.

**Table 2:** Demographic and clinical characteristics of the participants.

Variables	Healthy Group (*n* = 5)	MCI-AD Group (*n* = 22)
Age (years, median (IQR))	68 (68, 72)	72 (69, 74)
Gender (female, *n* (%))	2 (40%)	12 (54.5%)
CSF amyloid β-42 (pg mL^−1^, median (IQR))	1346.74 (930, 1421)	517.16 (453.86, 634.45)
CSF amyloid β-42/amyloid β-40 (median, IQR)	0.1 (0.09, 0.11)	0.05 (0.05, 0.05)
CSF *t*-Tau (pg mL^−1^, median (IQR))	240 (238, 276)	566 (450, 780)
CSF *p*-Tau (pg mL^−1^, median (IQR))	35 (35, 40)	81 (64.5, 107)
CSF NfL (pg mL^−1^, median (IQR))	826.94 (791, 847.7)	1428.68 (1123.24, 1555.91)
CSF *t*-Tau/amyloid β-42 (median (IQR))	0.2 (0.19, 0.25)	0.99 (0.79, 1.32)
CDR (score, median (IQR))	0 (0–0.5)	0.5 (0–1)
MMSE (score, median (IQR))	29 (29, 30)	24 (23, 26)
RBANS_DM (score, median (IQR))	100 (98, 110)	44 (40, 64)
FAQ (score, median (IQR))	1 (0, 2)	7 (4, 9)

CSF: cerebrospinal fluid; IQR: inter-quartile range; CDR: Clinical Dementia Rating; MMSE: Mini-Mental State Examination; RBANS_DM: The Repeatable Battery for the Assessment of Neuropsychological Status_Delayed Memory; FAQ: Functional Activities Questionnaire.

**Table 3:** Predicted target genes related to fatty acids for the selected miRNAs (miRBase).

miRNA	Target Genes
hsa-miR-494-3p	ELOVL3 (ELOVL fatty acid elongase 3)
	ELOVL5 (ELOVL fatty acid elongase 5)
hsa-miR-6894-3p	
hsa-miR-421	ARV1 (ARV1 homolog, fatty acid homeostasis modulator)
	FAR1 (fatty acyl-CoA reductase 1)
	ELOVL2 (ELOVL fatty acid elongase 2)
hsa-let-7a-3p	ELOVL2 (ELOVL fatty acid elongase 2)
	FA2H (fatty acid 2-hydroxylase)
	ELOVL7 (ELOVL fatty acid elongase 7)
hsa-miR-664a-3p	FAR1 (fatty acyl-CoA reductase 1)
	ELOVL4 (ELOVL fatty acid elongase 4)
	ELOVL7 ELOVL fatty acid elongase 7
	ELOVL5 ELOVL fatty acid elongase 5
hsa-miR-329-3p	
hsa-miR-450b-5p	ELOVL6 (ELOVL fatty acid elongase 6)
hsa-miR-323a-3p	
hsa-miR-382-5p	
hsa-miR-199b-3p	
hsa-miR-654-5p	FADS6 (fatty acid desaturase 6)
	ELOVL1 (ELOVL fatty acid elongase 1)
hsa-miR-2110	ELOVL4 (ELOVL fatty acid elongase 4)
hsa-miR-432-5p	
hsa-miR-505-3p	ELOVL4 (ELOVL fatty acid elongase 4)
hsa-miR-29a-3p	ELOVL4 (ELOVL fatty acid elongase 4)
hsa-miR-19b-3p	ELOVL5 (ELOVL fatty acid elongase 5)
hsa-miR-185-5p	ELOVL4 (ELOVL fatty acid elongase 4)
	ELOVL2 (ELOVL fatty acid elongase 2)
	FAR1 (fatty acyl-CoA reductase 1)
hsa-miR-576-5p	FAR2 (fatty acyl-CoA reductase 2)
hsa-miR-877-5p	
hsa-miR-29b-3p	ELOVL4 (ELOVL fatty acid elongase 4)
hsa-miR-143-3p	FADS6 (fatty acid desaturase 6)
	FAR1 (fatty acyl-CoA reductase 1)
hsa-miR-7976	
hsa-miR-5010-5p	
hsa-miR-4433b-5p	
hsa-miR-4433a-3p	FABP7 (fatty acid binding protein 7)
	ELOVL4 (ELOVL fatty acid elongase 4)
	ELOVL2 (ELOVL fatty acid elongase 2)

## Data Availability

The data that support the findings of this study are available on request from the corresponding author (C.C.-P.).

## Supplementary Materials

The following are available online at https://www.mdpi.com/article/10.3390/biomedicines9121812/s1, Figure S1: Correlation circle plot between miRNAs and lipids selected on each component, Figure S2: Boxplots representing lipid levels in participants’ groups, Table S1: Correlation matrix representing the individual correlation between miRNAs and lipids, Table S2: Median values for individual miRNAs and lipids in control and MCI-AD participants.
