# Supplementary material for: Epigenomics and Lipidomics Integration in Alzheimer Disease: Pathways Involved in Early Stages
Source: Biomedicines. 2021 Dec 2;9(12):1812. doi: 10.3390/biomedicines9121812 (PMC8698767; doi:10.3390/biomedicines9121812)
Supplement: Supplementary file 1 [file biomedicines-09-01812-s001.zip › biomedicines-1462788 supplementary revised.pdf]

Table S1. Correlation matrix representing the individual correlation between miRNAs and lipids.

	FA (20:2)	LPC (18:0)	LPE (18:0)	TG (16:0/20:4/18:1)	MG (22:4)	Cer (18:1/24:0)	FA (14:0)	FA (16:0)	FA (16:0)	FA (18:0)	FA (18:0)	FA (18:1)	FA (18:2)	FA (18:2)	FA (20:0)	FA (20:3)	FA (20:4)	PC (18:0/20:3)	PC (38:2)	PC (40:4)	PE (34:2)	PE (36:4)	PE (38:4)	PI (36:4)	TG (17:0/17:0/17:0)
**hsa-miR-29b-3p**	0.465	0.514	0.509	0.470	0.470	0.548	0.590	0.600	0.629	0.575	0.614	0.463	0.604	0.623	0.626	0.677	0.639	0.443	0.473	0.462	0.489	0.467	0.490	0.448	−0.564
**hsa-miR-664a-3p**	0.534	0.589	0.584	0.539	0.539	0.628	0.676	0.688	0.721	0.660	0.704	0.531	0.693	0.715	0.718	0.777	0.733	0.509	0.543	0.530	0.560	0.536	0.562	0.514	−0.647
**hsa-miR-2110**	0.516	0.570	0.565	0.521	0.522	0.608	0.655	0.666	0.698	0.638	0.682	0.514	0.671	0.692	0.695	0.752	0.709	0.492	0.525	0.513	0.542	0.518	0.544	0.497	−0.626
**hsa-miR-19b-3p**	0.456	0.503	0.499	0.460	0.460	0.536	0.577	0.587	0.616	0.563	0.601	0.453	0.592	0.610	0.613	0.663	0.626	0.434	0.464	0.452	0.478	0.457	0.480	0.439	−0.552
**hsa-miR-432-5p**	0.521	0.575	0.570	0.526	0.526	0.613	0.660	0.671	0.704	0.644	0.687	0.518	0.676	0.697	0.701	0.758	0.715	0.496	0.530	0.517	0.547	0.523	0.549	0.501	−0.631
**hsa-miR-323a-3p**	0.299	0.331	0.328	0.302	0.303	0.352	0.380	0.386	0.405	0.370	0.395	0.298	0.389	0.401	0.403	0.436	0.411	0.285	0.305	0.297	0.314	0.301	0.315	0.288	−0.363
**hsa-miR-329-3p**	0.518	0.572	0.566	0.522	0.523	0.609	0.656	0.667	0.699	0.640	0.683	0.515	0.672	0.693	0.696	0.753	0.711	0.493	0.527	0.514	0.543	0.519	0.545	0.498	−0.627
**hsa-miR-494-3p**	0.467	0.516	0.511	0.471	0.472	0.550	0.592	0.602	0.631	0.577	0.616	0.465	0.607	0.625	0.629	0.680	0.642	0.445	0.475	0.464	0.491	0.469	0.492	0.450	−0.566
**hsa-miR-654-5p**	0.515	0.569	0.563	0.519	0.520	0.606	0.652	0.664	0.696	0.636	0.679	0.512	0.668	0.689	0.693	0.749	0.707	0.490	0.524	0.511	0.541	0.517	0.542	0.496	−0.624
**hsa-miR-382-5p**	0.484	0.535	0.530	0.488	0.489	0.570	0.613	0.624	0.654	0.598	0.639	0.482	0.629	0.648	0.651	0.705	0.665	0.461	0.493	0.481	0.508	0.486	0.510	0.466	−0.587
**hsa-miR-5010-5p**	0.499	0.551	0.546	0.504	0.505	0.587	0.633	0.644	0.675	0.617	0.659	0.497	0.648	0.668	0.672	0.727	0.686	0.476	0.508	0.496	0.524	0.501	0.526	0.481	−0.605
**hsa-miR-4433b-5p**	0.469	0.518	0.513	0.473	0.474	0.552	0.594	0.605	0.634	0.580	0.619	0.467	0.609	0.628	0.631	0.683	0.644	0.447	0.477	0.466	0.493	0.471	0.494	0.452	−0.568
**hsa-miR-4433a-3p**	0.469	0.518	0.513	0.473	0.474	0.552	0.594	0.605	0.634	0.580	0.619	0.467	0.609	0.628	0.631	0.683	0.644	0.447	0.477	0.466	0.493	0.471	0.494	0.452	−0.568
**hsa-miR-185-5p**	0.506	0.559	0.553	0.510	0.511	0.595	0.641	0.652	0.684	0.625	0.668	0.503	0.657	0.677	0.681	0.736	0.695	0.482	0.515	0.502	0.531	0.508	0.533	0.487	−0.613
**hsa-let-7a- 3p**	0.510	0.564	0.558	0.515	0.516	0.600	0.647	0.658	0.690	0.631	0.673	0.508	0.663	0.683	0.687	0.743	0.701	0.486	0.519	0.507	0.536	0.512	0.538	0.491	−0.618

**hsa-miR-7976**	0.354	0.391	0.387		0.357	0.357		0.416	0.448	0.456	0.478	0.437	0.467	0.352	0.459	0.474	0.476	0.515	0.486		0.337	0.360	0.351	0.372	0.355	0.373	0.341		-0.429
**hsa-miR-576-5p**	0.509	0.562	0.557		0.514	0.514		0.599	0.645	0.656	0.688	0.629	0.672	0.507	0.661	0.682	0.685	0.741	0.699		0.485	0.518	0.505	0.535	0.511	0.536	0.490		-0.617
**hsa-miR-143-3p**	0.443	0.489	0.485		0.447	0.448		0.521	0.562	0.571	0.599	0.548	0.585	0.441	0.575	0.593	0.596	0.645	0.609		0.422	0.451	0.440	0.465	0.445	0.467	0.427		-0.537
**hsa-miR-877-5p**	0.465	0.514	0.509		0.470	0.470		0.547	0.590	0.600	0.629	0.575	0.614	0.463	0.604	0.623	0.626	0.677	0.639		0.443	0.473	0.462	0.489	0.467	0.490	0.448		-0.564
**hsa-miR-29a-3p**	0.510	0.563	0.558		0.515	0.515		0.600	0.646	0.657	0.689	0.630	0.673	0.507	0.662	0.683	0.686	0.742	0.700		0.486	0.519	0.506	0.536	0.512	0.537	0.491		-0.618
**hsa-miR-199b-3p**	0.403	0.445	0.441		0.407	0.407		0.474	0.511	0.519	0.544	0.498	0.532	0.401	0.523	0.539	0.542	0.586	0.553		0.384	0.410	0.400	0.423	0.404	0.424	0.388		-0.488
**hsa-miR-6894-3p**	0.504	0.557	0.552		0.509	0.510		0.593	0.639	0.650	0.681	0.623	0.665	0.502	0.655	0.675	0.678	0.734	0.692		0.480	0.513	0.501	0.529	0.506	0.531	0.486		-0.611
**hsa-miR-421**	0.541	0.598	0.592		0.546	0.547		0.637	0.686	0.697	0.731	0.669	0.714	0.538	0.703	0.724	0.728	0.788	0.743		0.516	0.550	0.537	0.568	0.543	0.570	0.521		-0.656
**hsa-miR-450b-5p**	0.531	0.586	0.580		0.535	0.536		0.624	0.672	0.684	0.717	0.656	0.700	0.528	0.689	0.710	0.714	0.772	0.729		0.505	0.540	0.527	0.557	0.532	0.559	0.511		-0.643
**hsa-miR-505-3p**	0.475	0.525	0.520		0.479	0.480		0.559	0.602	0.612	0.642	0.587	0.627	0.473	0.617	0.636	0.639	0.692	0.652		0.453	0.483	0.472	0.499	0.477	0.500	0.457		-0.576

Table S2. Median values for individual miRNAs and lipids in control and MCI-AD participants.

Analyte	Control (n=5) (Median,IQR)	MCI-AD (n=22) (Median, IQR)
hsa-miR-29b-3p	0 (0.0)	0 (0.25)
hsa-miR-664a-3p	0 (0.14)	0 (0.0)
hsa-miR-2110	0 (0.38)	0 (0.28)
hsa-miR-19b-3p	0 (0.12)	0 (0.59)
hsa-miR-432-5p	0 (0.19)	0 (0.56)
hsa-miR-323a-3p	0 (0.47)	0 (0.0)
hsa-miR-329-3p	0 (0.1)	0 (0.0)
hsa-miR-494-3p	0 (0.1)	0 (0.0)
hsa-miR-654-5p	0 (0.0)	0 (0.0)
hsa-miR-382-5p	0 (0.94)	0.5 (0.32)
hsa-miR-5010-5p	0 (0.1)	0 (0.37)
hsa-miR-4433b-5p	122 (109,334)	398 (160.864)
hsa-miR-4433a-3p	122 (109,334)	398 (160.864)
hsa-miR-185-5p	178 (126,237)	146 (63,281)
hsa-let-7a-3p	0 (0.21)	0 (0.3)
hsa-miR-7976	0 (0.44)	0 (0.0)
hsa-miR-576-5p	0 (0.0)	0 (0.0)
hsa-miR-143-3p	93 (32,1235)	271 (60.650)
hsa-miR-877-5p	0 (0.65)	0 (0.55)
hsa-miR-29a-3p	70 (17,182)	15 (0.130)
hsa-miR-199b-3p	10 (0.241)	36 (12,95)
hsa-miR-6894-3p	0 (0.0)	0 (0.2)
hsa-miR-421	0 (0.0)	0 (0.13)
hsa-miR-450b-5p	0 (0.1)	0 (0.0)
hsa-miR-505-3p	0 (0.25)	3 (0.17)
FA (20:0)	1.0 (0.7,1.4)	0.9 (0.7,1.3)
LPC (18:0)	0.5 (0.3,1.3)	0.3 (0.1,1.2)
LPE (18:0)	1.0 (0.8,1.6)	1.2 (0.6,2.1)
TG (16:0/20:4/18:1)	0.9 (0.9,1.0)	0.8 (0.7,1.0)
MG (22:4)	0.6 (0.4,1.6)	0.5 (0.3,0.7)
Cer (18:1/24:0)	1.8 (0.6,2.5)	1.7 (1.0.2.2)
FA (14:0)	1.1 (0.4,2.0)	0.9 (0.4,1.7)
FA (16:0)	1.1 (0.5,2.2)	0.8 (0.5,1.3)
FA (16:0)	1.1 (0.4,3.1)	0.7 (0.3,1.5)
FA (18:0)	1.1 (0.6,1.9)	0.8 (0.6,1.2)
FA (18:0)	1.2 (0.4,3.0)	0.7 (0.4,1.
FA (18:1)	1.1. (0.6,3.4)	0.9 (0.5,1.6)
FA (18:2)	0.8 (0.5,2.6)	1 (0.5,1.4)
FA (18:2)	0.6 (0.3,4.2)	1 (0.2,2.0)
FA (20:2)	1.0 (0.5,3.2)	0.9 (0.5,1.6)

FA (20:3)	1.1 (0.7,2.4)	1 (0.7,1.5)
FA (20:4)	1.1 (0.8,2.3)	1 (0.7,1.5)
PC (18:0/20:3)	1.1 (0.9,1.3)	1 (0.8,1.3)
PC (38:2)	1.1 (0.9,1.3)	1.1 (0.7,1.3)
PC (40:4)	0.8 (0.6,1.2)	0.8 (0.5,1.3)
PE (34:2)	0.7 (0.5,0.8)	0.6 (0.3,1.1)
PE (36:4)	0.8 (0.5,1.0)	0.5 (0.3,1.2)
PE (38:4)	1.2 (0.9,1.4)	0.8 (0.6,1.7)
PI (36:4)	1.1 (0.8,1.2)	0.9 (0.6,1.5)
TG (17:0/17:0/17:0)	1.2 (0.9,1.2)	1.3 (1.1,1.3)

IQR: Inter-quartile range
