# Supplementary figures and images for: Epigenomics and Lipidomics Integration in Alzheimer Disease: Pathways Involved in Early Stages
Source: Biomedicines. 2021 Dec 2;9(12):1812. doi: 10.3390/biomedicines9121812 (PMC8698767; doi:10.3390/biomedicines9121812)

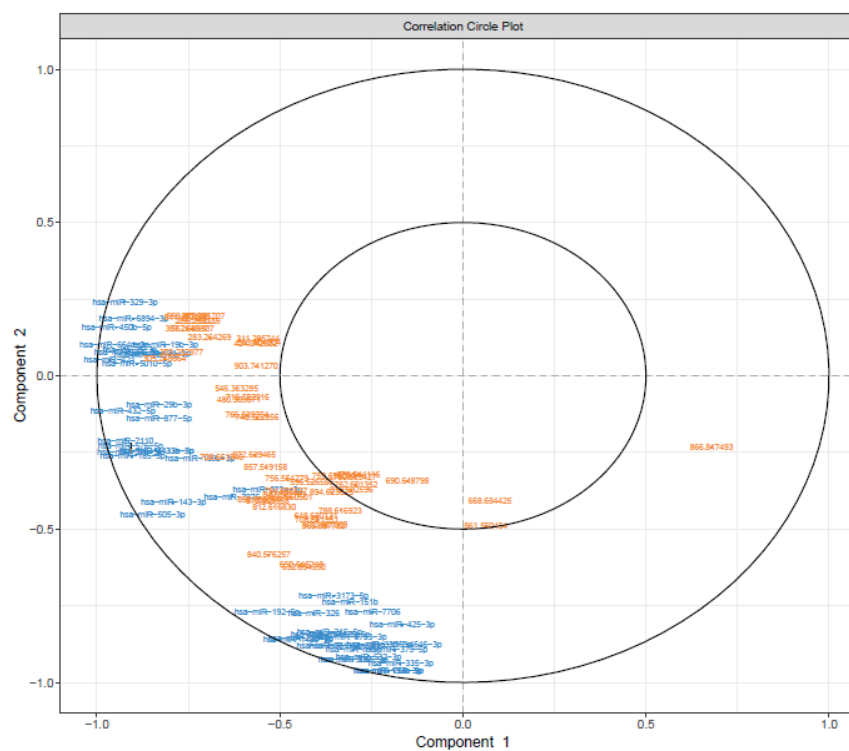

Figure S1. Correlation circle plot between miRNAs and lipids selected on each component.

Supplement: Supplementary file 1 [file biomedicines-09-01812-s001.zip › Figure S1.pdf]

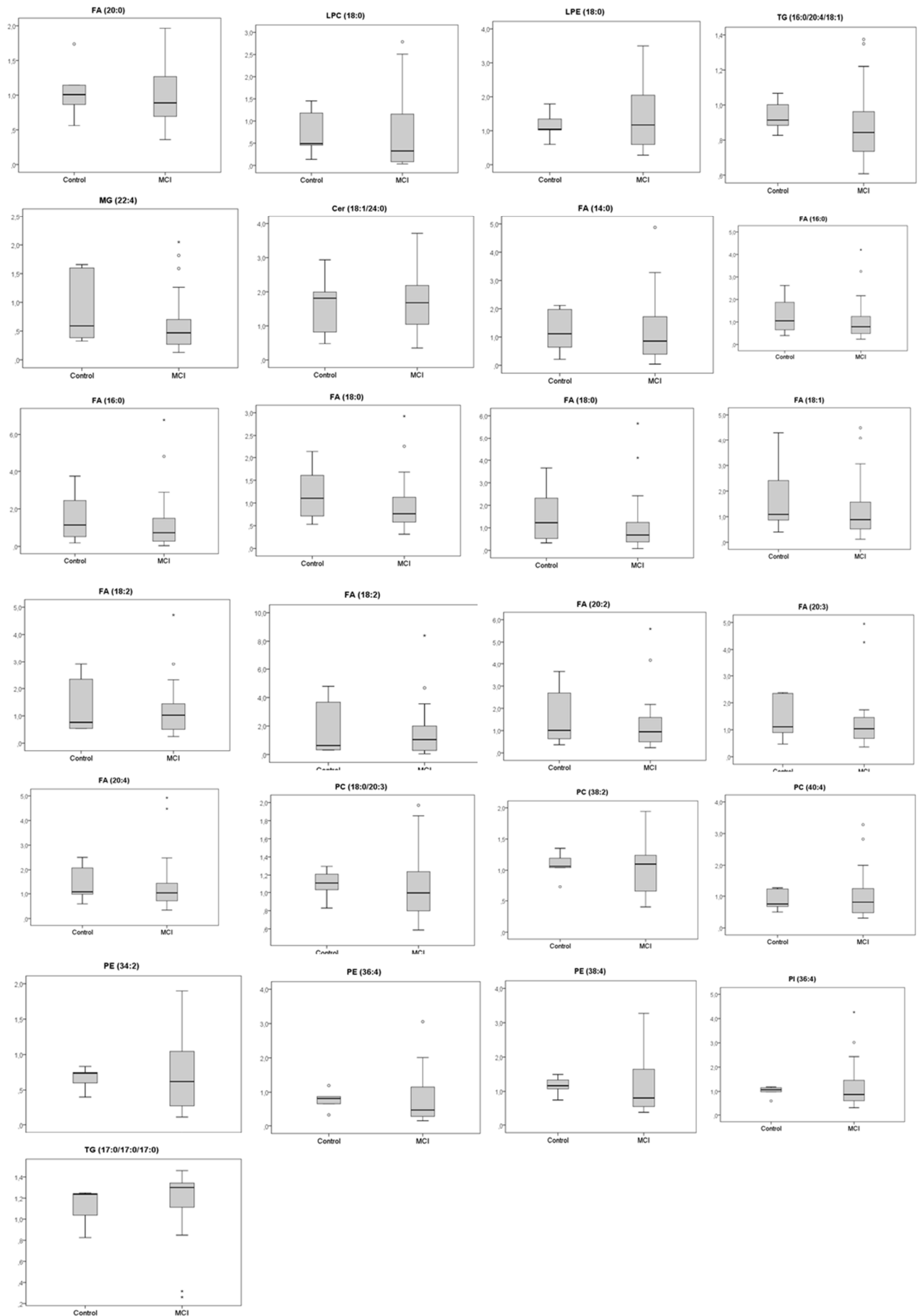

Figure S2. Boxplots representing lipid levels in participants' groups.

Supplement: Supplementary file 1 [file biomedicines-09-01812-s001.zip › Figure S2.pdf]
